# Supplementary material for: Acupuncture for Post-Operative Pain Relief and Functional Improvement in Tibial Fracture: A Systematic Review and Meta-Analysis
Source: Healthcare (Basel). 2025 Nov 12;13(22):2883. doi: 10.3390/healthcare13222883 (PMC12652893; doi:10.3390/healthcare13222883)
Supplement: Supplementary file 1 [file healthcare-13-02883-s001.zip › Table S6.pdf]

Supplementary Table S6. Leave-one-out sensitivity analysis for ROM

Study excluded	Mean difference	Heterogeneity
WANG 2020	MD: 2.09 [0.06, 4.12]<br>(P = 0.04)	$I^2 = 98\%$<br>(P < 0.00001)
ZHANG 2020	MD: 2.26 [0.51, 4.00<br>(P = 0.01)]	$I^2 = 97\%$<br>(P < 0.00001)
LONG 2021	MD: 1.08 [0.29, 1.87]<br>(P = 0.008)	$I^2 = 87\%$<br>(P = 0.0005)
XIAO 2022	MD: 1.78 [-0.24, 3.81]<br>(P = 0.08)	$I^2 = 98\%$<br>(P < 0.00001)

MD: Mean difference
