# Supplementary material for: Acupuncture for Post-Operative Pain Relief and Functional Improvement in Tibial Fracture: A Systematic Review and Meta-Analysis
Source: Healthcare (Basel). 2025 Nov 12;13(22):2883. doi: 10.3390/healthcare13222883 (PMC12652893; doi:10.3390/healthcare13222883)
Supplement: Supplementary file 1 [file healthcare-13-02883-s001.zip › Table S5.pdf]

Supplementary Table S5. Leave-one-out sensitivity analysis for HSS

Study excluded	Mean difference	Heterogeneity
LIU 2015	MD: 14.03 [9.53, 18.54]<br>(P < 0.00001)	I^2 = 93%<br>(P < 0.00001)
WANG 2020	MD: 14.01 [9.39, 18.63]<br>(P < 0.00001)	I^2 = 95%<br>(P < 0.00001)
FAN 2022	MD: 13.24 [8.36, 18.12]<br>(P < 0.00001)	I^2 = 95%<br>(P < 0.00001)
CHEN 2023	MD: 12.37 [8.09, 16.65]<br>(P < 0.00001)	I^2 = 94%<br>(P < 0.00001)
DENG 2024	MD: 14.45 [10.19, 18.70]<br>(P < 0.00001)	I^2 = 95%<br>(P < 0.00001)
LIU 2017	MD: 12.19 [8.03, 16.34]<br>(P < 0.00001)	I^2 = 94%<br>(P < 0.00001)
SI 2018	12.17 [8.15, 16.19]<br>(P < 0.00001)	I^2 = 93%<br>(P < 0.00001)

MD: Mean difference
