# Supplementary material for: Acupuncture for Post-Operative Pain Relief and Functional Improvement in Tibial Fracture: A Systematic Review and Meta-Analysis
Source: Healthcare (Basel). 2025 Nov 12;13(22):2883. doi: 10.3390/healthcare13222883 (PMC12652893; doi:10.3390/healthcare13222883)
Supplement: Supplementary file 1 [file healthcare-13-02883-s001.zip › Table S3.pdf]

Supplementary Table S3. The details of condition of participants

Author(year)	Sample size (I/C)	Condition
Deng 2024	30/30	Closed tibial plateau fracture in postoperative rehabilitation period<br>I: Schatzker type Ⅰ/Ⅱ/Ⅲ/Ⅳ (6/8/7/9)<br>C: Schatzker type Ⅰ/Ⅱ/Ⅲ/Ⅳ (7/9/7/7)
Chen 2023	30/30	Tibial plateau fracture in postoperative rehabilitation period<br>I: Schatzker type Ⅰ/Ⅱ/Ⅲ/Ⅳ/Ⅴ/Ⅵ (3/10/10/4/2/1)<br>C: Schatzker type Ⅰ/Ⅱ/Ⅲ/Ⅳ/Ⅴ/Ⅵ (4/9/8/5/3/1)
Wu 2023	20/18	Closed tibial plateau fracture in postoperative rehabilitation period<br>I: Not presented<br>C: Not presented
Quoc 2022	30/30	Tibial fractures in postoperative rehabilitation period (intramedullary nailing surgery)<br>I: Proximal third (3), middle third (10), distal third (14)<br>C: Proximal third (7), middle third (11), distal third (12)
Xiao 2022	36/36	Tibial plateau fracture in postoperative rehabilitation period<br>I: Schatzker type Ⅰ/Ⅱ/Ⅲ/Ⅳ (8/12/13/3)<br>C: Schatzker type Ⅰ/Ⅱ/Ⅲ/Ⅳ (7/13/14/2)
Fan 2022	46/46	Tibial fractures in postoperative rehabilitation period<br>I: Tibial shaft fracture/tibial plateau fracture (25/21)<br>C: Tibial shaft fracture/tibial plateau fracture (24/22)
Long 2021	73/73	Simple closed tibial plateau fracture in postoperative rehabilitation period<br>I: Schatzker type Ⅰ/Ⅱ/Ⅲ/Ⅳ/Ⅴ (20/15/16/17/5)<br>C: Schatzker type Ⅰ/Ⅱ/Ⅲ/Ⅳ/Ⅴ (19/16/18/14/6)
Cheng 2021	46/45	Closed tibial fractures in postoperative rehabilitation period<br>I: Proximal third (11), middle third (20), distal third (4), tibial plateau fracture (11)<br>C: Proximal third (10), middle third (19), distal third (5), tibial plateau fracture (11)
Zhang 2020	40/40	Tibial plateau fracture in postoperative rehabilitation period (open reduction internal fixation surgery)<br>I: Schatzker type Ⅰ/Ⅱ/Ⅲ/Ⅳ (8/13/15/4)<br>C: Schatzker type Ⅰ/Ⅱ/Ⅲ/Ⅳ (6/16/13/5)
Wang 2020	35/35	Tibial fractures in postoperative rehabilitation period<br>I: not presented<br>C: not presented
Wang 2019	50/50	Closed tibial plateau fracture in postoperative rehabilitation period<br>I: not presented<br>C: not presented
Zhang 2018	37/37	Tibial plateau fracture in postoperative rehabilitation period<br>I: not presented<br>C: not presented
Liu 2018	45/45	Tibial plateau fracture in postoperative rehabilitation period<br>I: not presented<br>C: not presented
Si 2018	53/52	Simple closed tibial fracture in postoperative rehabilitation period (Internal fixation using plates)<br>I: Transverse fracture (32), oblique fracture (17), spiral fracture (4); displaced fracture (23), non-displaced fracture (30); proximal third (16), middle third (26), distal third (11); Schatzker type Ⅰ/Ⅱ/Ⅲ (24/16/13)<br>C: Transverse fracture (30), oblique fracture (17), spiral fracture (5); displaced fracture

		(24), non-displaced fracture (28); proximal third (15), middle third (27), distal third (10); Schatzker type Ⅰ/Ⅱ/Ⅲ (22/17/13)
Liu 2017	53/38	Closed tibial fracture in postoperative rehabilitation period (internal fixation using plates)
		I: Transverse fracture (32)，oblique fracture (21), spiral fracture (6); proximal third (17), middle third (26), distal third (10); displaced fracture (23), non-displaced fracture (30); Schatzker type Ⅰ/Ⅱ/Ⅲ (21/15/17)
		C: Transverse fracture (24)，oblique fracture (14); proximal third (12), middle third (20), distal third (6); displaced fracture (12), non-displaced fracture (26); Schatzker type Ⅰ/Ⅱ/Ⅲ (10/18/10)
Liu 2015	43/43	Closed tibial plateau fracture in postoperative rehabilitation period (fixation using screws and plates)
		I: Schatzker type Ⅰ/Ⅱ/Ⅲ/Ⅳ/Ⅴ/Ⅵ (9/7/8/10/6/3)
		C: Schatzker type Ⅰ/Ⅱ/Ⅲ/Ⅳ/Ⅴ/Ⅵ (10/7/6/7/9/4)

The numbers in parentheses indicate the number of patients with the respective type of fracture. I: intervention group; C: control group
