# Supplementary material for: Acupuncture for Post-Operative Pain Relief and Functional Improvement in Tibial Fracture: A Systematic Review and Meta-Analysis
Source: Healthcare (Basel). 2025 Nov 12;13(22):2883. doi: 10.3390/healthcare13222883 (PMC12652893; doi:10.3390/healthcare13222883)
Supplement: Supplementary file 1 [file healthcare-13-02883-s001.zip › Table S9.pdf]

Supplementary Table S9. Leave-one-out sensitivity analysis for incidence of complications after surgery.

Study excluded	Odds ratio	Heterogeneity
LIU 2015	0.13 [0.06, 0.28] (P < 0.00001)	I^2 = 0% (P = 0.99)
ZHANG 2018	0.13 [0.06, 0.27] (P < 0.00001)	I^2 = 0% (P = 0.98)
LIU 2018	0.13 [0.06, 0.27] (P < 0.00001)	I^2 = 0% (P = 0.98)
WANG 2019	0.14 [0.06, 0.29] (P < 0.00001)	I^2 = 0% (P = 0.99)
WANG 2020	0.12 [0.05, 0.26] (P < 0.00001)	I^2 = 0% (P = 0.99)
XIAO 2022	0.11 [0.05, 0.25] (P < 0.00001)	I^2 = 0% (P = 0.99)
FAN 2022	0.12 [0.06, 0.26] (P < 0.00001)	I^2 = 0% (P = 0.98)
CHEN 2023	0.13 [0.06, 0.28] (P < 0.00001)	I^2 = 0% (P = 0.99)

OR: odds ratio
