# Supplementary material for: Acupuncture for Post-Operative Pain Relief and Functional Improvement in Tibial Fracture: A Systematic Review and Meta-Analysis
Source: Healthcare (Basel). 2025 Nov 12;13(22):2883. doi: 10.3390/healthcare13222883 (PMC12652893; doi:10.3390/healthcare13222883)
Supplement: Supplementary file 1 [file healthcare-13-02883-s001.zip › Table S2.pdf]

Supplementary Table S2. Detailed search strategy

Search strategy used in Cochrane library

NO.	Search strategy	Item
#1	MeSH descriptor: [Tibial Fractures] explode all trees	546
#2	(segond fracture*):ti,ab,kw OR (tillaux fracture*):ti,ab,kw OR (toddler fracture*):ti,ab,kw OR (tibia fracture*):ti,ab,kw	1283
#3	#1 OR #2	1516
#4	MeSH descriptor: [Acupuncture] explode all trees	217
#5	MeSH descriptor: [Electroacupuncture] explode all trees	1173
#6	(acupunctur*):ti,ab,kw OR (pharmacopunctur*):ti,ab,kw OR (pharmacoacupunctur*):ti,ab,kw OR (acupotom*):ti,ab,kw OR (acupoint*):ti,ab,kw OR (electroacupunctur*):ti,ab,kw OR (acupoint inject):ti,ab,kw OR (needling*):ti,ab,kw	26400
#7	#4 OR #5 OR #6	26400
#8	MeSH descriptor: [Randomized Controlled Trial] explode all trees	34
#9	("randomized controlled trial") OR ("controlled clinical trial") OR rct* OR placebo* OR random*	1587348
#10	#8 OR #9	1587348
#11	#3 AND #7 AND #10	8

Search strategy used in PubMed

NO.	Search strategy	Item
#1	"Tibial Fractures"[Mesh]	17,653
#2	"Segond Fracture*"[TW] OR "Tillaux Fracture*"[TW] OR "Fracture, Toddler*"[TW] OR "Tibia fracture*"[TW]	2,783
#3	#1 OR #2	18,620
#4	Acupuncture[Mesh]	32,179
#5	Electroacupuncture[MeSH]	5,496
#6	Acupunctur*[TW] OR Pharmacopunctur*[TW] OR Pharmacoacupunctur*[TW] OR Acupotom*[TW] OR Acupoint*[TW] OR Electroacupunctur*[TW] OR "Acupoint inject*"[TW] OR Needling*[TW]	43,391
#7	#4 OR #5 OR #6	44,691
#8	(("Controlled Clinical Trials as Topic"[Mesh] OR "Randomized Controlled Trials as Topic"[Mesh] OR Randomized Controlled Trial[PT] OR Controlled Clinical Trial[PT] OR Multicenter Study[PT] OR "Clinical Trials as topic"[Mesh] OR Random-Allocat*[TW] OR randomized[TW] OR randomised[TW] OR ((Double*[TW] OR single*[TW] OR treb*[TW] OR tripl*[TW]) AND (Blind*[TW] OR mask*[TW])) OR controlled-clinical-trial*[TW] OR controlled-trial*[TW] OR placebo*[TW] OR randomly*[TW]) NOT (Case Reports[PT] OR Letter[PT] OR meta-analys*[TW] OR metaanalys*[TW] OR "Meta-Analysis" [PT] OR case-report*[TW] OR Letter*[TI] OR "Systematic Review"[PT] OR Systematic-Review*[TI]))	1,878,702
#9	#3 AND #7 AND #8	2

Search strategy used in EMBASE

NO.	Search strategy	Item
#1	'tibia fracture'/exp	23,882
#2	'segond fracture*':ab,ti,kw OR 'tillaux fracture*':ab,ti,kw OR 'fracture, toddler*':ab,ti,kw OR 'tibial fracture*':ab,ti,kw	5,857
#3	#1 OR #2	24,656
#4	'acupuncture'/exp	62,771
#5	'electroacupuncture'/exp	10,631
#6	acupunctur*:ab,ti,kw OR pharmacopunctur*:ab,ti,kw<br>OR pharmacoacupunctur*:ab,ti,kw OR acupotom*:ab,ti,kw OR acupoint*:ab,ti,kw<br>OR electroacupunctur*:ab,ti,kw OR 'acupoint inject*':ab,ti,kw<br>OR needling*:ab,ti,kw	54,055
#7	#4 OR #5 OR #6	70,600
#8	('randomized controlled trial'/exp OR [randomized controlled trial]/lim<br>OR 'controlled clinical trial'/de OR 'multicenter study'/exp OR 'randomization'/exp<br>OR 'random allocat*':ab,ti,kw OR randomized:ab,ti,kw OR randomised:ab,ti,kw<br>OR 'controlled clinical trial*':ab,ti,kw OR 'controlled trial*':ab,ti,kw<br>OR placebo*:ab,ti,kw OR randomly*:ab,ti,kw OR<br>(((double* OR single* OR treb* OR tripl*) NEAR/3 (blind* OR mask*)):ab,ti,kw))<br>NOT ([conference abstract]/lim OR [conference paper]/lim OR [conference review]/lim OR [data papers]/lim OR [editorial]/lim OR [erratum]/lim OR [letter]/lim OR [note]/lim OR [review]/lim)	1,608,800
#9	#3 AND #7 AND #8	5

Search strategy used in China National Knowledge Infrastructure (CNKI)

NO.	Search strategy	Item
#1	SU='胫骨'*'骨折'	23,044
#2	SU='针灸'+'针刺'+'acupuncture'+'electroacupuncture'+'pharmacoacupuncture'+'acupotomy'+'acupoint'+'needling'	239,444
#3	#1 AND #2	50

Search strategy used in Japan Science and Technology Information Aggregator Electronic (J-stage)

NO.	Search strategy	Item
#1	Full text: tibial fracture AND Full text: acupuncture AND Full text: random	6
#2	Full text: 脛骨 AND Full text: 骨折 AND Full text: 針 AND Full text: 臨床	26

Search strategy used in Research Information Service System (RISS)

NO.	Search strategy	Item
#1	전체 : 경골 <AND> 전체 : 골절 <AND> 전체 : 침	2
#2	전체 : tibial <AND> 전체 : fracture <AND> 전체 : acupuncture	3
#3	전체 : tibial <AND> 전체 : fracture <AND> 전체 : needle	4

Search strategy used in Korean Studies Information Service System (KISS)

NO.	Search strategy	Item
#1	전체 ="경골" and 전체 ="골절" and 전체 ="침"	1
#2	전체 ="tibial" and 전체 ="fracture" and 전체 ="acupuncture"	0
#3	전체 ="tibial" and 전체 ="fracture" and 전체 ="needle"	1

Search strategy used in Oriental Medicine Advanced Searching Integrated System (OASIS)

NO.	Search strategy	Item
#1	경골 AND 골절 AND 침	1
#2	tibial AND fracture AND acupuncture	2
#3	tibial AND fracture AND needle	0
