# Supplementary material for: Acupuncture for Post-Operative Pain Relief and Functional Improvement in Tibial Fracture: A Systematic Review and Meta-Analysis
Source: Healthcare (Basel). 2025 Nov 12;13(22):2883. doi: 10.3390/healthcare13222883 (PMC12652893; doi:10.3390/healthcare13222883)
Supplement: Supplementary file 1 [file healthcare-13-02883-s001.zip › Table S4.pdf]

Supplementary Table S4. Leave-one-out sensitivity analysis for VAS

Study excluded	Mean difference	Heterogeneity
CHENG 2021	MD: -0.85 [-1.21, -0.49] (P < 0.00001)	I^2 = 64% (P = 0.04)
QUOC 2022	MD: -1.09 [-1.55, -0.63] (P < 0.00001)	I^2 = 87% (P < 0.0001)
FAN 2022	MD: -1.00 [-1.65, -0.34] (P = 0.003)	I^2 = 88% (P < 0.0001)
WU 2022	MD: -1.02 [-1.47, -0.57] (P < 0.00001)	I^2 = 88% (P < 0.0001)
DENG 2024	MD: -1.19 [-1.57, -0.81] (P < 0.00001)	I^2 = 76% (P = 0.006)

MD: Mean difference
