# Supplementary material for: Acupuncture for Post-Operative Pain Relief and Functional Improvement in Tibial Fracture: A Systematic Review and Meta-Analysis
Source: Healthcare (Basel). 2025 Nov 12;13(22):2883. doi: 10.3390/healthcare13222883 (PMC12652893; doi:10.3390/healthcare13222883)
Supplement: Supplementary file 1 [file healthcare-13-02883-s001.zip › Table S8.pdf]

Supplementary Table S8. Complications after surgery

Author (year)	Acupuncture group	Control group
Chen 2023	NP	NP
Xiao 2022	post-traumatic arthritis (1), venous thrombosis (1)	malunion (1), post-traumatic arthritis (2), venous thrombosis (2), joint stiffness (4)
Fan 2022	joint stiffness (1)	venous thrombosis (2), joint stiffness (4)
Wang 2020	post-traumatic arthritis (1), joint stiffness (1)	malunion (1), post-traumatic arthritis (3), venous thrombosis (2), joint stiffness (3)
Wang 2019	0*	NP
Liu 2018	NP	NP
Zhang 2018	nonunion (1)	malunion (3), nonunion (3), venous thrombosis (1)
Liu 2015	0*	NP

The numbers in parentheses indicate the number of individuals with the respective complication. *No individuals with the respective complication. NP: not presented. *
