# Supplementary material for: Acupuncture for Post-Operative Pain Relief and Functional Improvement in Tibial Fracture: A Systematic Review and Meta-Analysis
Source: Healthcare (Basel). 2025 Nov 12;13(22):2883. doi: 10.3390/healthcare13222883 (PMC12652893; doi:10.3390/healthcare13222883)
Supplement: Supplementary file 1 [file healthcare-13-02883-s001.zip › Table S7.pdf]

Supplementary Table S7. Leave-one-out sensitivity analysis for effective rate

Study excluded	Odds ratio	Heterogeneity
LIU 2017	5.58 [2.91, 10.71] ($P < 0.00001$)	$I^2 = 0\%$ ($P = 0.97$)
SI 2018	4.76 [2.59, 8.74] ($P < 0.00001$)	$I^2 = 0\%$ ($P = 0.95$)
WANG 2020	4.82 [2.63, 8.83] ($P < 0.00001$)	$I^2 = 0\%$ ($P = 0.94$)
LONG 2021	4.95 [2.58, 9.50] ($P < 0.00001$)	$I^2 = 0\%$ ($P = 0.94$)
FAN 2022	4.97 [2.77, 8.94] ($P < 0.00001$)	$I^2 = 0\%$ ($P = 0.94$)
XIAO 2022	4.93 [2.69, 9.03] ($P < 0.00001$)	$I^2 = 0\%$ ($P = 0.94$)
DENG 2024	4.58 [2.54, 8.26] ($P < 0.00001$)	$I^2 = 0\%$ ($P = 0.98$)

OR: odds ratio
